# Community seroprevalence of hepatitis B, C and human immunodeficiency virus in adult population in gojjam zones, northwest Ethiopia

**DOI:** 10.1186/s12985-017-0696-6

**Published:** 2017-02-06

**Authors:** Bayeh Abera, Yesuf Adem, Mulat Yimer, Wondemagegn Mulu, Yohannes Zenebe, Zewdie Mekonnen

**Affiliations:** 1 Department of Microbiology, Immunology & Parasitology, College of Medicine & Health Sciences, Bahir Dar University, Bahir Dar, Ethiopia; 2 Department of Biochemistry, College of Medicine & Health Sciences, Bahir Dar University, Bahir Dar, Ethiopia; 3 Department of Biomedical research, Biotechnology Research Institute, Bahir Dar University, Bahir Dar, Ethiopia

**Keywords:** Community, HBV, HCV, HIV

## Abstract

**Background:**

In Ethiopia, there is lack of data on the prevalence of hepatitis B virus (HBV), hepatitis C virus (HCV) and human immune deficiency virus (HIV) infections in adult population at community level. This study aimed at determining the HBV, HCV and HIV seroprevalence in adult population at community level in East and West Gojjam zones in Amhara region, Ethiopia.

**Methods:**

A cross-sectional study was conducted between October 01 and November 30, 2015. The Hepatitis B surface antigen (HBsAg) and anti-HCV were detected using the standard serological tests. The antibody to HIV infection was tested using the national HIV rapid tests algorithms.

**Results:**

A total of 481 adults comprised of 51% females with median age of 25 years took part in the study. Overall, 7.5% (95% CI: 5.5–10.2%) of adult population were infected either with HBV, HCV and HIV. The prevalence of HBV was 15 (3.1%) and for HIV was 16 (3.3%). The seroprevalence of HCV was five (1.0%). HIV-HCV co-infection was found to be two (0.4%). HIV prevalence was higher in non-educated population than their counter parts (*P* = 0.001). HIV prevalence was high in housewives (6.0%) and merchants (4.7%).

**Conclusions:**

This study revealed an intermediate HBV prevalence and low prevalence of HCV in adult population at community level. HIV prevalence is still a major public health problem in the area. To have the national data, we recommend further study on genotypes of HBV and HCV including local risk factors for transmissions. Moreover, health education on HBV, HCV and HIV transmission should be an intervention measure in the community.

## Backgrounds

Infection with HBV, HCV and HIV are still a major public health problem worldwide, particularly in developing countries [1]. HBV and HCV infections can cause both acute and chronic liver disease, including cirrhosis and hepatocellular carcinoma (HCC). Studies showed that 80% of HCC was caused by active replication of chronic HBV and HCV infections [2, 3]. For instance, about 686, 000 people die every year due to complications of HBV infection (cirrhosis and HCC) [4].

According to the World Health Organization (WHO) report, 34 million people are HIV positive worldwide [5]. In Ethiopia, the prevalence of HIV infection differs significantly among regions and different population groups. WHO estimated that 753,100 people are living with HIV in Ethiopia [6, 7]. However, the national HIV prevalence declined from 1.5 to 1.1% in 2015 [6, 7]. Urban populations are more affected with HIV than rural areas while females are twice affected than male population [6]. Globally, about 400 million and 170 million people are chronically infected with HBV and HCV, respectively [8]. Approximately 1 million people die each year from hepatitis B and C virus infections [9].

In Ethiopia, there is lack of information on the prevalence of HBV, HCV and HIV in the general population. This is due to the fact that virtually all prevalence data on HBV, HCV and HIV have been obtained in selected group of people with risk factors such as pregnant women, military personnel and patients with liver disease [10–12]. For instance, the prevalence of HBV and HCV in pregnant women was between 4.4 and 0.26%, respectively [13]. Therefore, the prevalence of HBV, HCV and HIV infections at the community level is not known. The present study was therefore conducted to determine the prevalence of HBV, HCV and HIV infections among adults at house hold level in the community in East and West Gojjam zones in Amhara region, Ethiopia.

## Methods

### Study design and period

We conducted a community-based cross sectional study in East and West Gojjam administrative zones in Amhara region between October 01 and November 30, 2015. From each household, only one adult who gave consent to participate in the study were involved in the study.

### Source and study population

The source population was all apparently healthy adult population in East and West Gojjam Zones in the Amhara region. Apparently healthy adults with age ≥ 18 years of both sexes from the selected households in the study area were deemed a study population.

### Sample size and sampling

The sample size was calculated using single population proportion formula. It was calculated by taking a proportion of 0.5 by assuming that the mean (μ) population for each parameter is known to be 50%, 2% marginal error, and 95% confidence levels. Therefore, the sample size of 481 was determined. The households from each district were selected using systematic random sampling technique after getting the nth value by dividing the total number of households with the total number of selected households.

### Data collection

Trained laboratory technicians interview study participants and collected data on demographic, monthly income, education and marital status were collected by face to face interview using a structured questionnaire.

### Detection of HBsAg and anti-HCV and anti-HIV

Trained laboratory technicians collected 5 ml of whole blood aseptically from vein by puncture. The collected blood was allowed to clot then serum was separated by centrifugation at room temperature. Antibody to HIV infection was tested using the national HIV rapid diagnostic tests algorithm. Initially, HIV infection was screened using KHB (Bio-Engineering, Shanghai, Kehua). The HIV positive samples were re-tested with STAT PAK (Chembio diagnostic, Newyork, USA). HIV positive serums which showed discordant results between the first test (KHB) and the second test (STAT PAK) were tested with Unigold (Trinity Biotech Bray, Ireland) as tie breaker.

The presence of hepatitis B surface antigens (HBsAg) in serum was detected using advanced quality One Step Rapid Test per instructions of the manufacturer (Zhejiang Orient Gene Biotech, Zhejiang, China). The anti-HCV antibody was detected using One Step Rapid Test per instructions of the manufacturer (Zhejiang Orient Gene Biotech, Zhejiang, China). The samples found to be positive for HBV and HCV had been re-tested in duplicate. The HIV positive samples were re-tested with STAT PAK per the manufacturers’ instruction. To ensure the reliability and quality of data, positive and negative control sera were run following the manufacturer recommendations alongside the test kits.

### Data analysis

Data was entered into Statistical Package for Social Science *(IBM Corp. Released 2011. IBM SPSS Statistic Armonk, NY: IBM Corp)*. A categorical variable were tested using the Chi-square tests. All statistical tests were two-tailed, and the significance level was set at *P* < 0.05.

### Ethical clearance

The Research Ethics Review Committee of the College of Medicine and Health Sciences of Bahir Dar University has approved the ethical clearance. Written consent was obtained from each study participants to provide blood for HBV, HCV and HIV determination for research purpose. The study participants found to be positive for HBV, HCV and HIV were reported to physicians and/or health officers for treatment and counselling. Information obtained at any course of the study was kept confidential.

## Results

### Socio-demographic profiles

A total of 481 adults comprised of 245 (51%) of females and 236 (49%) males from the community participated in the study. The median age of the study participants was 25 years (ranged 18–60 years). Assessment of education showed that 153 (31.8%) of the participants did not have formal education. The majority (97.1%) of participants were urban residents. Table 1 depicts the marital status, monthly income and occupation of the study participants.

**Table 1:** Socio-demographic profiles of study participants for HBV, HCV and HIV

Variables		Number	Percentage
Sex	Female	245	51.0
	Male	236	49.0
Age group	18–25 years	257	53.5
	>26 years	224	46.5
Residence	Urban	467	97.0
	Rural	14	3.0
Education	Elementary and above	351	73.0
	No formal education	130	27.0
Marital status	Single	245	51.0
	Married	189	39.3
	Windowed	20	4.2
	Divorced	27	5.5
Monthly income	<500 birr	324	67.4
	≥500 birr	157	32.6
Occupation	Government employee	105	21.8
	Merchant	64	13.3
	Housewife	85	17.7
	Farmer	35	7.3
	Non-employee	191	39.7
Total		481	100

### Prevalence of HBV, HCV and HIV infection

Overall, 7.5% (95% CI: 5.5–10.2%) of adult population at the community were infected either with hepatitis B or C viruses and HIV. Of these, HBV prevalence accounted for 3.1%. The seroprevalence of HCV was 5 (1.0%). The prevalence of HIV was 3.3%. In this study, only two (0.4%) adults were co-infected with HCV and HIV. However, HBV and HIV co-infection was not found. Table 2 illustrated the prevalence of HBV, HCV and HIV infections.

**Table 2:** Seroprevalence of HBV, HCV and HIV among adult population

Variables	Positive		Negative	Total
	N (%)	95%, CI	N (%)	
HBsAg	15 (3.1)	1.9, 5.1	466 (96.9)	481 (100)
Ant-HCV antibody	5 (1.0)	0.4, 2.4	476 (99)	481 (100)
Anti-HIV antibody	16 (3.3)	2.1, 5.33	465 (96.3)	481 (100)
Co-infection (HIV&HCV)	2 (0.4)	0.1, 1.5	479 (87.5)	481 (100)
Total	36 (7.5)	5.5,10.2	445 (92.5)	481 (100)

### Risk factor for HBV, HCV and HIV prevalence

In the present study, no statistical significant differences were observed for HBV and HCV infections in terms of age, sex, residence and marital status. However, HIV prevalence was in higher in females than males (*P* = 0.001). Furthermore, HIV prevalence was higher in non-educated population than their counter parts (*P* = 0.001) (Table 3). Considering occupations of adults, HIV prevalence was higher in merchants (4.7%) than others.

**Table 3:** Association of HBV, HCV and HIV prevalence with sociodemogrphic variables

Variables	HBsAg N (%)		*P*- value	HIV status N (%)		*P* value	HCV status N (%)		*P* value
	Pos	Neg		Pos	Neg		Pos	Neg	
Sex									
Females	7 (3.0)	238 (97)		12 (5.0)	233 (95)	0.001	3 (1.2)	242 (98.8)	NA
Males	8 (3.5)	228 (96.5)	0.92	4 (1.7)	232 (98.3)		2 (0.8)	233 (99.2)	
Age group									
18–25 years	8 (3.2)	249 (96.8)	0.93	8 (3.0)	249 (97.0)		2 (0.8)	255 (99.2)	NA
≥26 years	7 (3.2)	217 (96.8)		8 (3.6)	216 (96.4)	0.48	3 (1.3)	221 (98.7)	
Residence									
Urban	14 (3.0)	453 (97)	0.62	15 (3.2)	452 (96.8)		5 (1.1)	462 (98.9)	
Rural	1 (7.7)	13 (92.3)		1 (8.2)	12 (92.8)	NA	–	13 (100)	NA
Marital status									
Single	8 (3.4)	237 (96.6)		8 (3.2)	237 (96.8)		2 (0.8)	243 (99.2)	
Married	6 (3.5)	183 (96.5)	0.95	6 (3.1)	183 (96.9)	NA	3 (1.6)	186 (98.4)	
Widowed	–	20 (100)	NA	-	20 (100)		–	20 (100)	NA
Divorced	1 (3.8)	26 (96.2)	NA	2 (7.4)	25 (92.6)		–	27 (100)	
Education									
Elementary and above	12 (3.4)	339 (96.6)	0.28	6 (1.7)	345 (98.3)	0.001	3 (0.8)	348 (99.2)	
No formal education	3 (2.3)	126 (97.7)		10 (7.8)	119 (92.2)		2 (1.6)	127 (98.4)	NA
Monthly income									
<500 birr	12 (3.7)	312 (96.3)	0.22	14 (4.3)	310 (95.7)	0.08	4 (1.2)	320 (98.8)	NA
≥500 birr	3 (1.9)	154		2 (1.3)	155 (98.7)		1 (0.6)	156 (99.4)	
Occupation									
Government employee	3 (2.8)	102 (97.2)	NA	3 (2.8)	102 (97.2)	NA	–	105 (100)	
Merchant	2 (3.1)	62 (96.9)		3 (4.7)	61 (95.3)		1 (1.6)	63 (98.4)	NA
Housewife	3 (3.5)	82 (97.2)		5 (6.0)	80 (94.0)		1 (1.2)	84 (98.8)	
Farmer	1 (2.8)	34 (97.2)		–	35 (100)		–	35 (100)	
Non-employee	6 (3.1)	185 (96.9)		5 (2.6)	186 (97.4)		3 (1.6)	188 (98.4)	

NA: not applicable

## Discussion

This study showed the prevalence of HBV, HCV and HIV infections among adult population at the community level. Overall, 7.5% of the adult population was infected either with hepatitis B or C viruses and HIV. This indicated that HBV, HCV and HIV infections are the major public health problems in Ethiopia. HIV prevalence in the present study (3.3%) is higher than the national adult HIV prevalence which is 1.1% [14]. Furthermore, HIV prevalence in females (5.2%) was higher than the national HIV prevalence among females (1.4%) [3]. Like other reports, the prevalence of HIV was significantly higher in females than males (*P* = 0.001) [14–16].

In this study, a statistically significant association was observed between HIV prevalence and educational levels. HIV prevalence was higher among non-educated adults (*P* = 0.001). Likewise, studies indicated that HIV prevalence in developing countries was higher among less educated groups [8, 17]. It is well documented that in the early stage of HIV pandemic, the prevalence of HIV infection was higher among well educated people in urban areas than non-educated people. However, in later stage, due to the availability of information on prevention of HIV transmission, it decreases in educated people [17]. Furthermore, the prevalence of HIV was higher in merchants. This conforms to a study conducted in Ethiopia which stated that merchants had higher odds of being HIV positive than others [18].

This study showed an intermediate (3.1%) prevalence of HBV in adults at community level compared to 5–10% HBV prevalence in adult population in sub-Saharan Africa [9]. Likewise, a study conducted in Libya reported 2.2% HBV prevalence in a general population [19]. In contrast, it is lower compared to other studies conducted in various part of Ethiopia. For instance, 3.8 to 4.4% of HBV prevalence was reported among pregnant women and military person [10, 13, 20]. A statistically significant association was not observed between HBV positive and risk factors. Although, it was not statistically significant, adults with low monthly income (<500 birr) had higher HBV prevalence (3.7 vs 1.9%) compared to those adults with better income (*P* = 0.22).

Hepatitis C virus (HCV) prevalence in adult population was low (1.0%). Similarly, low prevalence (0.6 to 0.7%) of HCV was reported in pregnant women [11, 21]. In contrast, higher prevalence of HCV was documented among attendants of voluntary counseling and testing for HIV in Ethiopia. For instance, 9.1, 6.0 and 4.3% HCV prevalence were reported in Hawassa, Mekelle and Adwa, respectively [22–24]. This clearly showed that HCV in the general population is low while higher in risk group of the population.

People with HIV infection are often co-infected with chronic viral hepatitis by HBV and HCV due to common modes of transmission. Two (0.4%) adults were co-infected by HCV and HIV. However, a study conducted on selected risk group of people reported higher co-infection of HIV-HBV (34%), HIV-HCV (8.0%) and HBV-HCV (2%) [25]. Moreover, 5.6% HIV-HBV and 5% HIV-HCV co-infections were documented in HIV positive individuals [26]. These differences might be due to the study population differences. The Quantative-real time polymerase chain reaction (qRT-PCR) was not used to confirm the positive samples. Therefore, this deemed to be a certain limitation for this study.

## Conclusion

This study showed an intermediate prevalence for HBV and low for HCV in adult population at community level. However, HIV prevalence was higher in non-educated adults at community levels. We recommend further national wide studies on the prevalence and genotypes of HBV and HCV including risk factors in the general population.

## Abbreviations

HbsAg: Hepatitis B surface antigen
HBV: Hepatitis B virus
HCV: Hepatitis C virus and HIV human immune deficiency virus
HHC: Hepatocellular carcinoma
